# Implementation of an antimicrobial stewardship program in a multicenter neonatal intensive care unit collaborative: a mixed-methods staff resource needs evaluation

**DOI:** 10.1038/s41372-026-02758-y

**Published:** 2026-06-22

**Authors:** Cynthia L. Gong, Nabeel Qureshi, Peter Mendel, Kurlen S. E. Payton, Henry C. Lee, Kenneth M. Zangwill

**Affiliations:** 1 Division of Neonatology, Department of Pediatrics, Children’s Hospital Los Angeles, Keck School of Medicine, Fetal and Neonatal Institute, University of Southern California, Los Angeles, CA USA; 2 RAND Corporation and Pardee RAND Graduate School, Santa Monica, CA USA; 3 RAND Corporation, Santa Monica, CA USA; 4 Division of Neonatology, Department of Pediatrics, Cedars-Sinai Medical Center, Los Angeles, CA USA; 5 Division of Neonatology, University of California San Diego, La Jolla, CA USA; 6 Division of Pediatric Infectious Diseases and The Lundquist Institute at Harbor-UCLA Medical Center, Torrance, CA USA

**Keywords:** Paediatrics, Outcomes research

## Abstract

**Objective:**

Staffing needs for an antimicrobial stewardship program (ASP) in the neonatal intensive care unit setting are undefined.

**Study design:**

In an ASP collaborative of 29 California NICUs, we surveyed sites to determine full-time equivalents (FTE) for ASP implementation effort by clinical role, clinical acuity, patient volume, and other NICU characteristics.

**Results:**

146 staff members performed ASP implementation, including neonatologists, nurses, hospitalists, nurse practitioners, pharmacists, information technology personnel, and infectious disease specialists. The 8-month preparation and 12-month intervention phases required a median (IQR) of 0.015 (0.011–0.025) and 0.071 (0.056–0.091) FTEs (0.6 and 2.8 hours per week), respectively. Neonatologists and nurses performed 43% and 19% of the workload, respectively; effort for site-level stewardship activities and collaborative learning dissemination varied 3.3-fold over the collaborative. Higher clinical acuity and patient volume were associated with higher neonatologist effort.

**Conclusion:**

Driven by neonatologists and nurses, NICU ASP requires demonstrable effort, and staff needs vary considerably.

## Introduction

Inpatient antimicrobial stewardship programs (ASPs) can be very effective towards optimizing clinical care and providing favorable economic outcomes in neonatal, pediatric and adult patient populations [1–4]. Given the large amount of data supporting such programs, many national organizations have called for dedicated infrastructural support and increased individual effort to support antibiotic stewardship [5, 6]. Such programs will vary in their intensity and breadth depending on a variety of factors specific to the institution, unit, available resources, and participating staff availability and interest.

Robust stewardship activities may involve a wide variety of participants, including physicians, advanced practice and other nurses, pharmacists, infection preventionists, and administrative personnel such as information technology and quality improvement professionals. ASPss are developed amid potentially complex organizational structures related to the targeted inpatient population (hospital-wide v individual units), local needs, and clinician willingness and availability. Despite calls for dedicated antimicrobial stewardship activities in U.S. hospitals, there is no data-driven information on the required minimum staffing level or effort individual institutions or units should support [7, 8].

The neonatal intensive care unit (NICU) has unique challenges with implementing ASPs, including provider- and institution-level barriers [9–11]. This is complicated by a lack of readily available pediatric-trained infectious disease doctors and pharmacists in many units outside of urban areas. Previous studies in the NICU have also highlighted the lack of access to formalized stewardship programs and activities targeted towards reduction in antibiotic use, and typically reflect practice from large volume, high acuity NICUs. Herein, we report a description of the staffing and effort burden required to enable an antibiotic stewardship program during a large multicenter collaborative in the state of California [11, 12].

## Methods

Data for this analysis were gathered as part of the Optimizing Antibiotic Stewardship in California NICUs (OASCN) collaborative (www.cpqcc.org/improvement/projects/OASCN), conducted within the California Perinatal Quality Care Collaborative (CPQCC) in partnership with the RAND Corporation, Lundquist Institute at Harbor-UCLA Medical Center, and University of Southern California. The collaborative included 29 NICU sites that participated in biweekly 60-min live virtual learning sessions focusing on a specific stewardship topic presented through patient case studies and didactic sessions, as well as discussion of monthly antibiotic use trends from participating NICUs, and participant feedback on strategies, challenges, and progress. These sessions were complemented by the distribution and availability of resources, including a newsletter summarizing each learning session, “Office Hours” on special topics, an email listserv, an online quality improvement (QI) data submission and analytics portal, and an archive containing Learning Session presentations, videos, and a curated reference library.

As part of the collaborative, a mixed-methods study was conducted to gather longitudinal data on participant perceptions and experiences and other drivers of implementation and stewardship outcomes [11]. The analysis presented here focuses on data collected from NICU site leaders through a monthly status survey, semi-structured pre- and post-intervention phone interviews, and check-in calls approximately every 3–4 months to assess site-level challenges, progress, and plans with implementing stewardship activities, and effort spent on collaborative activities by site staff above and beyond their usual clinical duties (Supplementary Data).

In particular, we employed this approach to gather information on the amount of time members of each NICU’s stewardship improvement team responsible for ASP implementation spent on different collaborative and site stewardship improvement activities over the course of the study. Types of NICU site personnel that we asked about included neonatologists, other prescribers (hospitalists, pediatricians and nurse practitioners), nurses (including nurse leaders [supervisors, managers, quality improvement and other nurse specialists] and bedside nurses), pharmacists (including clinical pharmacy coordinators), information technology (IT) staff and infectious disease specialists. The data collected were classified into two time periods: preparatory activities after site recruitment began but before the collaborative kickoff (15 June 2020–19 February 2021) and the “intensive collaborative” intervention year of the project (1 March 2021–28 February 2022).

Within each period, we categorized time by specific ASP implementation activities, which included stewardship improvement team members preparing for and attending collaborative learning sessions, facilitating participation of staff in the stewardship improvement team and/or collaborative learning sessions, dissemination of collaborative learning among other site staff, site-level stewardship improvement activity, and data reporting. The NICU site leaders were asked to provide details on who was involved in each activity type and approximately how much time each individual spent on these activities. The pre-intervention phone interviews were administered 1–2 months prior to the start of the collaborative, and during the last 10 weeks of the “intensive collaborative” intervention year [11]. Respondents were asked to estimate effort by all members of a particular personnel type in the unit (e.g., neonatologists).

To quantify the time investment in terms of full-time equivalents (FTEs), we documented the estimated number of hours spent on each activity, as detailed by the NICU site leaders. These hours were then converted into FTE days per week, assuming a standard 40-hour work week. By extrapolating FTE days, we calculated the total number of FTEs for the entire year of the collaborative, as well as FTEs per 1000 patient-days. These estimations encompassed both preparatory activities and intervention period activities to provide a comprehensive understanding of the staffing and time required for collaborative and ASP implementation.

Using these estimations, we calculated the median FTE effort and hours/week required by each personnel type to conduct the collaborative and stewardship improvement activities, stratified by various key NICU characteristics based on 2020 data collected by CPQCC. Key characteristics included American Academy of Pediatrics (AAP) level of care (levels 2, 3, and 4 in our sample) [13], California Children’s Services level (community, intermediate, regional, and non-CCS) [14], and tertile ranges for total annual number of live births, NICU admissions, NICU patient-days, NICU days with antibiotic exposure, and number of NICU beds. Tertile ranges were calculated using all CPQCC member NICUs (*n* = 135), which were then applied to each of the OASCN participating sites (*n* = 29). Cutoffs for each tertile are shown in Table 1. We compared FTEs by personnel type within the preparatory and intervention phases using ANOVA, while FTEs by hospital characteristics were compared using Kruskal-Wallis chi-square tests. FTEs by ASP implementation activity type (e.g., attending collaborative learning sessions, disseminating collaborative learning to other site staff, site-level stewardship improvement activities) were compared using the Wilcoxon signed rank test. All statistical analyses were conducted in R [15].

**Table 1:** NICU Characteristics Across All CPQCC Member Sites (2020), by Tertile Range.

Characteristic	Tertile Ranges		
	1st	2nd	3rd
Total live births^a^	538–1662	1663–2873	2874–7081
Total NICU admits	9–229	230–365	366–1188
Total NICU patient-days	59–2551	2552–5593	5594–27,217
Antibiotic exposure (patient-days)	5–319	320 - 834	835–6096
Number of NICU beds	2–15	16–24	25–104

Tertile ranges were calculated based on 2020 data using all CPQCC member NICUs (*n* = 135), which were then applied to each of the sites that participated in the OASCN collaborative. All OASCN analyses were stratified using these ranges.

^a^The first tertile range for this variable excludes five Level 4 NICUs that only admit patients through transfers from lower level NICUs, and thus report zero live births.

This work was approved by the RAND Human Subjects Protection Committee, the Stanford Panel on Medical Human Subjects, and the John F. Wolf Human Subjects Committee of The Lundquist Institute, in accordance with the Declaration of Helsinki. All participants provided consent to participate in interviews with oral consent. The datasets generated and analyzed during the current study are not publicly available due to other ongoing analyses and manuscript writing in progress using the same data. They are, however, available from the corresponding author upon reasonable request.

## Results

In the cohort of 29 NICUs completing the OASCN collaborative, 4 (14%), 21 (72%), and 4 (14%) were AAP level 2, 3, and 4 units, respectively. By CCS level, 19 (66%) were community, 2 (7%) intermediate, and 6 (21%) regional (two were non-CCS). The distribution of other characteristics is described in Table 1.

Participants with an active role in site stewardship improvement teams and ASP implementation activities during the OASCN collaborative are enumerated by clinical role in Table 2. During the intervention phase, the 29 OASCN sites collectively reported 146 staff who were members of a participating site stewardship improvement team. In this one year period, sites reported involvement of 59 neonatologists (40% of all site stewardship improvement staff), 40 nurses (30 of whom were nurse leaders and 10 bedside nurses; total 28%), 21 pharmacists (14%), 8 information technology (IT) personnel (5%), 9 other prescribers (hospitalists and nurse practitioners; total 6%), and 5 infectious disease specialists (3%). During the 8-month preparation period, 112 staff were involved during which there were slightly more “other prescribers” (7% vs. 6%) participating than in the intervention phase, but less participation by pharmacists and slightly less so by neonatologists. Participation in ASP implementation activities by nurse leaders was similar in the preparatory and intervention phases; 26 of 29 sites (90%) had nurse leaders involved in the intervention year. Participation of bedside nurses in ASP implementation activities, however, was much lower than nurse leaders. Only 3 of the 29 sites reported having bedside nurses involved in the preparatory phase, although it doubled to 6 sites by the intervention phase. Three (3) sites did not report any nurse (nurse leader, bedside, or QI specialist) involvement in either phase.

**Table 2:** Distribution of Stewardship Improvement Team Staff by Clinical Role.

Clinical Role	Preparatory Phase Total *N* (min, max per NICU)	Intervention Phase Total *N* (min, max per NICU)
Neonatologist	43 (1,4)	59 (1,5)
Nurse	39 (0,4)	44 (0,4)
Pharmacist	9 (0,1)	21 (0,3)
Other prescribers	10 (1,2)	9 (0,2)
IT staff	8 (1,2)	8 (0,2)
Infectious Disease Specialists	3 (0,1)	5 (0,1)
**TOTAL**	**112**	**146**

^*^Nurse category includes nurse leaders, bedside nurses, and nurse QI specialists. Note that three sites did not report nurse participation in their site stewardship improvement team. 31 nurse leaders were involved in their site stewardship improvement team during the Preparatory phase, and 29 in the Intervention phase. During the Preparatory phase, 4 sites reported having a total of 7 nurses and 1 nurse QI specialist involved. During the intervention phase, 6 of the 29 sites reported having bedside nurses (and at least 1 nurse leader), while 4 reported having one nurse QI specialist involved. This represents 8 total individual nurses in the Preparatory phase, and 13 total individual nurses in the Intervention phase.

** Other prescribers category includes hospitalists and nurse practitioners; no sites reported involvement of physician assistants in their stewardship improvement team.

Across the two project phases, the preparation period required total staffing of a median of 0.015 FTEs (IQR 0.011–0.025) and the intervention period required 0.071 FTEs (IQR 0.056–0.091), or an overall median of 0.58 and 2.83 h per week, respectively (Table 3). During the intervention period, neonatologists performed the largest proportion of ASP implementation effort, a median of 0.039 FTEs (1.54 hours/week). This represented 54% of the median expended effort for ASP implementation effort (2.83 h/week); significantly more than each of the other work types (*p* < 0.02). Other personnel types include other prescribers (nurse practitioners and/or hospitalists, 0.015 FTEs, 0.6 h), nurses (0.014, 0.5 h), IT staff (0.011, 0.5 h), pharmacists (0.008, 0.3 h), and infectious disease specialists (0.004, 0.2 h). Importantly, there was substantial variation in ASP implementation effort across the 29 reporting sites, with interquartile ranges varying to different degrees, dependent on the clinical roles involved in stewardship improvement activities in a given NICU.

**Table 3:** Median full time equivalent work effort, by project phase and clinical role.

Clinical Role	Median FTE Effort	25%,75% IQR^a^	Median Hours/week	25%,75% IQR
	**8 Month Preparatory Phase**			
Neonatologist	0.005	0.004, 0.011	0.19	0.16, 0.45
Nurse	0.004	0, 0.007	0.14	0, 0.29
Pharmacist	0	0, 0.004	0	0, 0.35
Other prescribers^b^	0.004	0, 0.006	0.14	0, 0.56
IT staff	0.006	0.005, 0.007	0.22	0.20, 0.66
Infectious Disease Specialist	0	0, 0.001	0	0, 0.14
**Entire cohort**^c^	**0.015**	**0.011, 0.025**	**0.58**	**0.43, 1.0**
	**12 Month Intervention Phase**			
Neonatologist^d^	0.039	0.025, 0.154	1.54	0.99, 6.16
Nurse	0.014	0.004, 0.073	0.54	0.18, 2.94
Pharmacist	0.008	0.003, 0.031	0.33	0.13, 1.23
Other prescribers^b^	0.015	0.007, 0.047	0.61	0.330, 1.88
IT staff	0.011	0.002, 0.028	0.45	0.06, 1.14
Infectious Diseases	0.004	0.002, 0.021	0.18	0.09, 0.83
**Entire cohort**	**0.071**	**0.056, 0.091**	**2.83**	**2.22, 3.62**

^a^IQR = inter-quartile range.

^b^Other prescribers in both the preparatory and intervention phases included hospitalists and nurse practitioners.

^c^Median FTE effort and median hours per week for the entire cohort are reported overall across staffing types, and are not equivalent to the sum of the median FTEs and hours reported for each clinical role.

^d^Neonatologists had a higher FTE effort than each of the other clinical roles (*p* < 0.02).

**Fig. 1:**
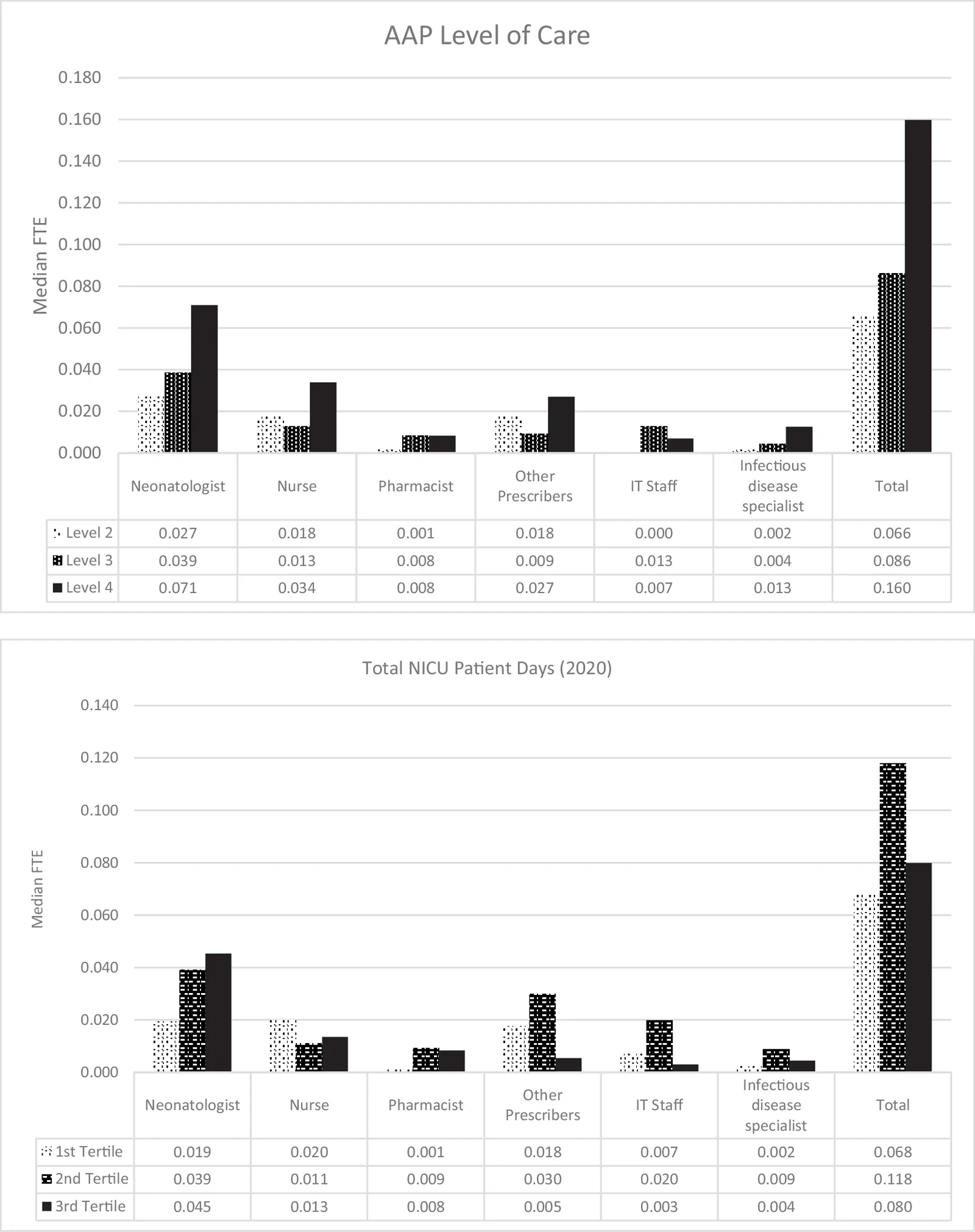
Intervention phase FTE effort by AAP level of care and patient days. Level 4 NICUs reported the highest level of effort across most staff types, except for IT staff. There was a general trend of increasing effort as the AAP level of care increased. When stratified by total number of NICU patient days, NICUs in the 2nd tertile (2252-5993 patient days) reported the highest total FTEs at 0.118 per week compared to those in the 1st tertile (0-2251 patient days) or 3rd tertile (5594-27,217 patient days).

**Fig. 2:**
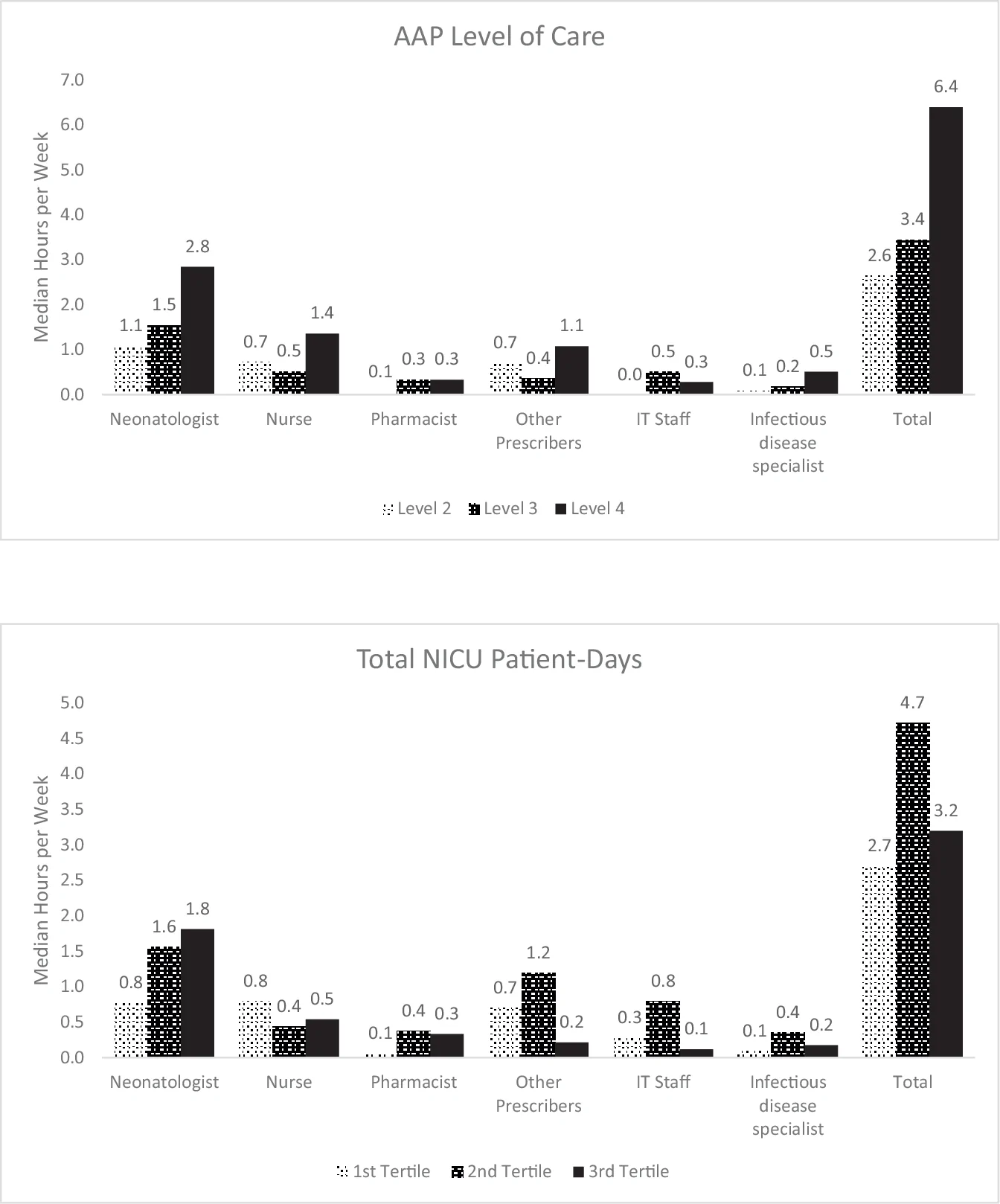
Median hours per week, by AAP level of care and patient-days. Hours per week effort increased with increasing AAP level of care; however, when stratified by total NICU patient days, total hours per week were highest among hospitals stratified in the 2nd tertile (2552-5593 patient days).

As illustrated in Fig. 1 and Fig. 2, FTE effort during the intervention period increased with increasing AAP level of care provided (*p* = 0.05). While level 4 NICUs expended more effort than Level 3 (median 0.16 and 0.086 FTEs respectively, driven by increases among neonatologists, prescribers, and infectious disease specialists. When analyzed by patient volume (patient-days), neonatologists’ FTE burden increased with volume (*p* = 0.04), but not so among the other clinical roles (Figs. 1 and 2). No trend was seen when NICUs were stratified by CCS level, total live births, total NICU admits, number of NICU days with antibiotic exposure, or number of NICU beds during the intervention period.

**Fig. 3:**
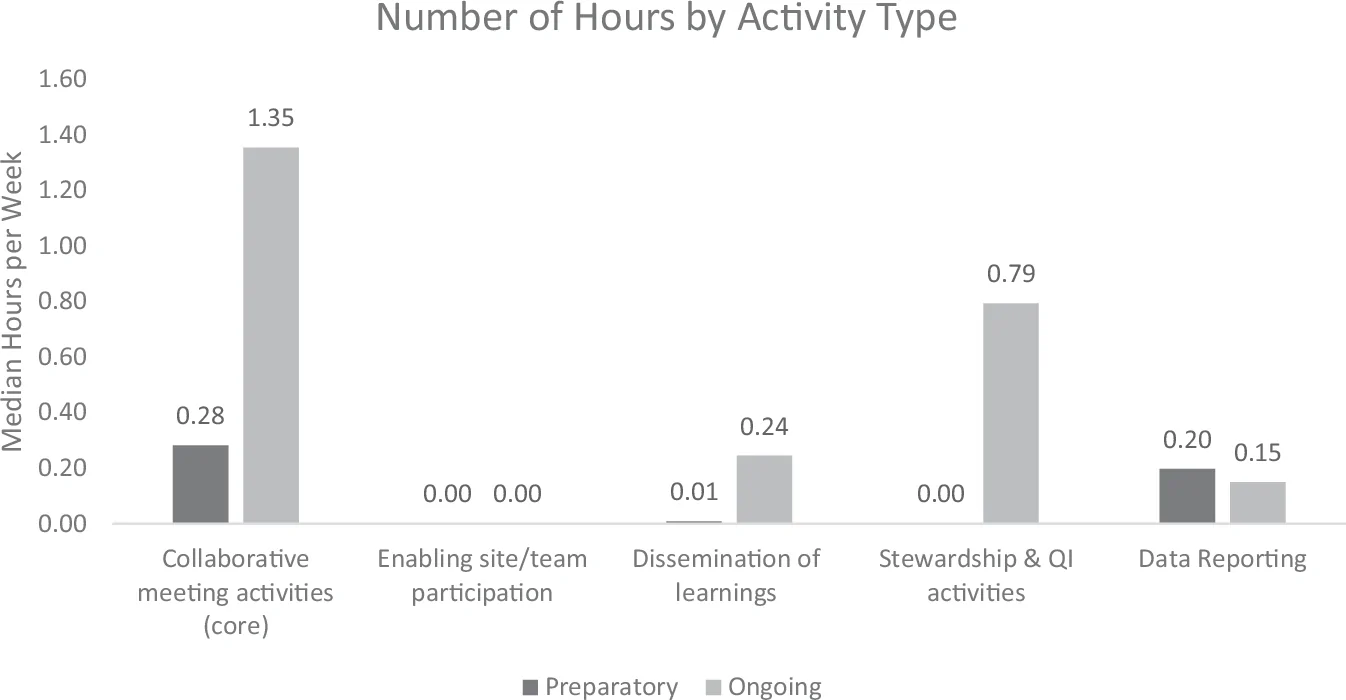
Median number of hours per week, by activity type. Hours per week are stratified by collaborative activity type, indicating that most time was spent on core collaborative meetings (e.g., educational sessions) and stewardship activities. The remaining amount of time was split between dissemination of learnings and data reporting.

In terms of FTEs associated with specific ASP-implementation activities during the intervention phase (Fig. 3), preparing for and attending collaborative learning sessions (median 0.034 [IQR 0.037–0.058] FTEs) and dissemination of learnings among staff and site-level stewardship improvement activities (0.02 [IQR 0.011–0.045] dominated; which translates into a median of 1.35 and 0.79 h/week, respectively. As with the overall ASP implementation effort by clinical role described above, there was variation in these efforts across the NICUs in the OASC collaborative. The interquartile range for median FTE effort specifically for site-level learning dissemination and stewardship improvement activities varied ~3.3-fold within the collaborative. Overall effort for site-level stewardship improvement and dissemination combined did not vary substantially by AAP level of care or NICU patient days.

## Discussion

We provide an analysis of human resource requirements for performing robust antimicrobial stewardship in the NICU setting; we are not aware of any similar data on this topic in the literature, for any target population or clinical unit. In our collaborative of 29 NICUs in California including a wide variety of unit types, populations, and geography within the state, the site ASP-implementation teams required a median of >7% of an FTE during the active intervention period and neonatologists accounted for more than one half of the reported necessary effort and the largest participating personnel type followed by nurses. Unsurprisingly, we found that pharmacists were actively involved in nearly three-quarters of our sites. Infectious disease specialists were not commonly utilized in contradistinction to general pediatric and adult inpatient settings, where they often play a prominent role [16]. This may reflect a specific decision at the unit level and/or potentially a lack of availability, as there are <2 pediatric infectious disease physicians per 100,000 U.S. children with very uneven distribution geographically [17]. Neonatologists and pharmacists are often the ASP “champion”, yet our data suggest the value of a broader multidisciplinary approach, such as including hospitalists and nurses. Universally recommended, such teams empower staff towards a culture of meaningful antibiotic stewardship [18, 19].

There are no specific data-driven staffing recommendations for ASPs in US hospitals, and none for the NICU specifically. We found a low overall median FTE requirement accounting for both preparatory and ongoing activities for our OASCN units, with higher reported needs depending on a variety of factors. In the only published evaluation of overall FTE effort, a survey of active ASP practitioners from 244 US hospitals (only 16% of whom took care of children) noted that an average (no median provided) of 0.88 FTEs was in force, but that another 0.38 FTEs would be desired for full effectiveness [20]. Others have proposed staffing requirements such as an expert Working Group in Canada (5 staff/1000 acute care general hospital beds) [21] and with time-in-motion data from the US Veterans Health Administration system (one pharmacist/100 beds) [22]. In our stratified analyses, increased AAP level of care (a proxy for increasing patient complexity) and patient volume corresponded with an increasing level of neonatologist effort, a trend seen in the Doernberg et al. survey as well [20].

We do not provide dollar cost estimates that would flow from the FTE estimates we developed. This necessarily depends on several factors, all local to the NICU and/or its healthcare system as applicable. Regardless, it is also true that typically those who lead NICU-based ASPs do not receive remuneration for this specific responsibility. This is likely due to several factors related to the relatively small population size of NICUs compared to the rest of a children’s hospital or community hospital in which the NICU is located, relatively fewer direct medical cost dollars potentially saved in this small population (although no data exist on this), and as previously reported, a lack of awareness of the value or need of such activities in the NICU, particularly in general community hospitals in which NICU needs may not be as understandable or even visible to decision-makers [11]. In this current environment, we encourage others to generate local FTE information to support a business case to implement ASP [23]. Further, as of January 2024, the Center for Medicare and Medicaid Services required critical access hospitals to attest to reporting antibiotic use to the Centers for Disease Control and Prevention’s National Healthcare Safety Network [24], potentially facilitating more administrative openness to providing ASP staff for NICUs.

The distribution of work by specific activity in part reflects the nature of the OASCN collaborative processes. The largest percentage of work in the intervention period was associated with the biweekly one-hour learning sessions, which provided continuous opportunities for group interactive learning (brief didactic sessions) and for sharing of implementation method successes and challenges experienced by individual NICUs (“office hours”), a formal qualitative assessment of which has been reported previously [11]. Yet, overt bedside stewardship and local dissemination of learnings took approximately the same effort as the learning sessions, but this varied quite a bit among the 29 participating units. The time required to complete the preparatory work was minimal (~1–2%), nearly all of which was done by neonatologists and IT staff.

The distribution of job types in a given ASP is dependent on available local expertise and clinician interest; this has not been described in the NICU setting in a robust fashion. In our cohort, nurse leaders contributed greatly to the ASP effort in nearly all NICUs and in nearly a third, bedside nurses actively participated. A 2018 national survey noted that >80% of neonatal advanced practice registered nurses (APRNs) are involved in quality improvement projects [25], but there are fewer APRNs in any given NICU than bedside nurses. We previously reported that APRNs in the OASCN cohort skewed towards the units with greater patient volume [12]. Several position statements call for active involvement of bedside nurses in ASP activities, and how they may specifically contribute [26], but as noted by the Agency for Healthcare Research and Quality in 2019, the “role of [bedside] nursing in stewardship interventions has largely been overlooked” [27]. We believe NICUs should actively explore ways to integrate all available and interested nursing staff into ASP efforts—a concept the American Nurses Association and CDC actively endorse as well [28].

Our cohort is generally representative of the distribution of US NICUs regarding clinical acuity, size, and populations served [29]. We believe our results, however, to be a conservative estimate of FTEs actually required. For example, our surveyors queried about work effort on a “per week” basis without further clarification, and our calculations assume a standard 40 h work week [30]. This does not necessarily reflect clinical practice, during which stewardship effort is a consideration every day of the week, for all patients. Also, our data reflect responses to phone interviews performed once during the preparatory phase and 2–3 times over the course of the intervention phase, as well as shorter check-in calls throughout the intervention phase, specifically focused on cost and FTE metrics. It is possible that ASP-associated work was not recalled or recognized by our respondents at the time of our interviews. Finally, the shared learning model among participants in the OASCN collaborative, in real time on video calls, may have facilitated ASP decision-making and therefore would not be “counted” by respondents.

Given the timing of the collaborative, we inquired about the impact of the ongoing COVID-19 pandemic on workflows. Most of the individuals listed as members of site stewardship improvement teams were those clinically present in the unit, so remote or hybrid work had minimal impact. Overall, site leaders reported issues with staffing, particularly of nursing staff, some reductions in census, and changes in operations to account for COVID-19-infected patients and mothers; however, most NICU leaders reported little to no impact of COVID-19 on stewardship activities. A few reported some initial issues meeting as a team to plan stewardship activities virtually, but this was not a significant barrier to planning and implementing stewardship improvement activities and was resolved as more meetings became virtual. There are no published data on the impact of COVID on the workforce needs related to antimicrobial stewardship in the NICU setting.

We acknowledge that these data are not without limitations - some of which may limit generalizability to other NICUs. First, collaborative stewardship activities may overlap with overlying hospital protocols and routine work; the true amount of time dedicated to ASP activities may have been confounded. Similarly, we had no way to externally validate OASCN-specific effort in units, relying on respondent recall for the specific amount of time spent per week on collaborative activities, independent of regular management-related activities. Also, it is possible that participation in the collaborative video calls every two weeks may have been considered actual stewardship (learnings) by participants and not explicitly labeled alternatively as local “stewardship and QI activities”, as queried. Our data are only from the state of California, potentially reflecting ASP principles that may differ from elsewhere; many basic ASP tenets, however, are unlikely to be incorporated differently by geography. Lastly, the CPQCC ran an ASP collaborative with a different set of NICUs 6 years before our work [31], but we believe the impact on our findings is minimal to none, given the intervening time period and, in particular, the COVID pandemic during which, indeed, decreased capacity for robust stewardship has been reported [32]. Nonetheless, we attempted to quantify stewardship FTE burden in a manner that may be useful for other institutions interested in implementing an ASP.

Our data, from a large and diverse statewide collaborative, provide the first estimate of the minimum FTE requirement for antimicrobial stewardship in the NICU setting. While neonatologists accounted for most of the active intervention work, nearly all participating NICUs employed a multidisciplinary team, the composition of which was determined by local needs and goals. There was also substantial variation observed between individual NICUs in work effort and stewardship team composition. Our findings can help inform NICU ASP programs as they are implemented (or reconsidered) with respect to the desired intensity and breadth of the effort.

## Data Availability

Upon request.
